# Comparing health-related quality of life in modified Rankin Scale grades: 15D results from 323 patients with brain arteriovenous malformation and population controls

**DOI:** 10.1007/s00701-021-04847-7

**Published:** 2021-04-16

**Authors:** Anni Pohjola, Elias Oulasvirta, Risto P. Roine, Harri P. Sintonen, Ahmad Hafez, Päivi Koroknay-Pál, Hanna Lehto, Mika Niemelä, Aki Laakso

**Affiliations:** 1 Department of Neurosurgery, Helsinki University Hospital, Topeliuksenkatu 5B, 00260 Helsinki, Finland; 2 Group Administration, University of Helsinki and Helsinki University Hospital, Helsinki, Finland; 3 Department of Health and Social Management, University of Eastern Finland, Kuopio, Finland; 4 Department of Public Health, University of Helsinki, Helsinki, Finland

**Keywords:** Arteriovenous malformation, Cerebrovascular malformations, Modified Rankin Scale, Quality of life

## Abstract

**Background:**

We wanted to understand how patients with different modified Rankin Scale (mRS) grades differ regarding their health-related quality of life (HRQoL) and how this affects the interpretation and dichotomization of the grade.

**Methods:**

In 2016, all adult patients in our brain arteriovenous malformation (AVM) database (*n* = 432) were asked to fill in mailed letters including a questionnaire about self-sufficiency and lifestyle and the 15D HRQoL questionnaire. The follow-up mRS was defined in 2016 using the electronic patient registry and the questionnaire data. The 15D profiles of each mRS grade were compared to those of the general population and to each other, using ANCOVA with age and sex standardization.

**Results:**

Patients in mRS 0 (mean 15D score = 0.954 ± 0.060) had significantly better HRQoL than the general population (mean = 0.927 ± 0.028), *p* < 0.0001, whereas patients in mRS 1–4 had worse HRQoL than the general population, *p* < 0.0001. Patients in mRS 1 (mean = 0.844 ± 0.100) and mRS 2 (mean = 0.838 ± 0.107) had a similar HRQoL. In the recently published AVM research, the most commonly used cut points for mRS dichotomization were between mRS 1 and 2 and between mRS 2 and 3.

**Conclusions:**

Using 15D, we were able to find significant differences in the HRQoL between mRS 0 and mRS 1 AVM patients, against the recent findings on stroke patients using EQ-5D in their analyses. Although the dichotomization cut point is commonly set between mRS 1 and 2, patients in these grades had a similar HRQoL and a decreased ability to continue their premorbid lifestyle, in contrast to patients in mRS 0.

## Introduction

Modified Rankin Scale (mRS) is a commonly used functional outcome instrument in neurological and neurosurgical research [39]. Our understanding of the grade itself has improved with the increasing use of the utility-weighted mRS (UW-mRS), which incorporates patient preferences into the outcome evaluation [8]. The utility weights have been determined using different health-related quality of life (HRQoL) instruments; however, to our knowledge, the 15D has not been used in these analyses. Compared to other instruments, 15D has been reported to be more sensitive for psychological and mental dimensions and has a lower ceiling effect than for instance EQ-5D [33, 38]. Secondly, dichotomization of the mRS has become popular, although it has its drawbacks [9]. The benefits include the easier analysis and interpretation of results, as well as lower error rates in interobserver variability [1, 22]. We wanted to deepen our understanding of the outcomes in each mRS class and investigate how dichotomization might affect research results. We used questionnaire and clinical data from 323 patients with brain arteriovenous malformation (AVM) to compare HRQoL measured with the 15D instrument in patients with different mRS grades and with age- and sex-standardized general population. We also performed a literature review of AVM research using mRS dichotomization. With the understanding of both the literature and our results, we discuss our hypothesis that mRS 0 forms a distinct group of patients, whose HRQoL outcomes are considerably better than those in the other mRS classes and that this should be taken into account in the interpretation of results.

## Materials and methods

The Helsinki AVM Database includes 805 patients with brain AVM admitted to the Helsinki University Hospital Department of Neurosurgery between the years 1942 and 2014. The database has been collected retrospectively using medical records and images. The questionnaire letters were mailed in 2016 to all adult (age > 18 years) patients (*n* = 432) in the database. The letter contained separate questions regarding symptoms, comorbidities, lifestyle, and self-sufficiency/independence, along with the 15D HRQoL questionnaire. Of those approached, 325 (75.2%) answered. There were only two patients with mRS 5, and they were excluded from the study, the final study cohort thus consisting of 323 patients. Patients were classified into mRS grades using the electronic patient registry and the self-sufficiency questionnaire. The classification was done after the patient had returned the mailed questionnaire.

### HRQoL measurement: 15D

HRQoL was measured by the generic self-administered 15D instrument. It can be used both as a profile and as a single index score measure. The questionnaire includes 15 dimensions: mobility, vision, hearing, breathing, sleeping, eating, speech, excretion, usual activities, mental functioning, discomfort and symptoms, depression, distress, vitality, and sexual activity. For each dimension, the respondent chooses one of the five ordinal levels best describing his/her state of health at the moment (best value = 1; worst value = 5) [36]. The single index score (15D score) represents the overall HRQoL on a 0–1 scale (1 = full health, 0 = being dead) and the dimension level values reflect the goodness of the levels relative to no problems on the dimension (= 1) and to being dead (= 0). They are calculated from the questionnaire using a set of population-based preference or utility weights. Mean dimension level values are used to draw 15D profiles for groups. A change or difference in the 15D score of ± 0.015 is clinically important [2]. Further properties of the instrument are described at http://15d-instrument.net/15d/.

### Statistical methods

Patients with missing data were excluded from the analysis of the variable or dimension in question. Two patients (0.9%) had not filled in the entire 15D questionnaire. They were included only in the dimension level analyses for the dimensions they had answered. The 15D data for the general population came from the National Health 2011 Survey representing the Finnish population aged over 18. For this analysis, those individuals were selected, who were from the Helsinki University Hospital catchment area and in the age range of patients (*n* = 1350). This sample was weighted to reflect the age and sex distribution of the patients, separately for each mRS grade [19]. The equality of the mean 15D scores across mRS grades was tested with ANCOVA (age and sex standardized), followed by Bonferroni corrected post hoc tests. The profiles comparing mRS grades to each other were drawn based on the estimated means after age and sex standardization. The *p* values < 0.05 were considered statistically significant. Statistical analysis was performed using the SPSS for Mac statistical software version 25 (SPSS, Inc., Chicago, IL, USA). The study adheres to STROBE reporting guidelines.

### Literature review

**Fig. 1:**
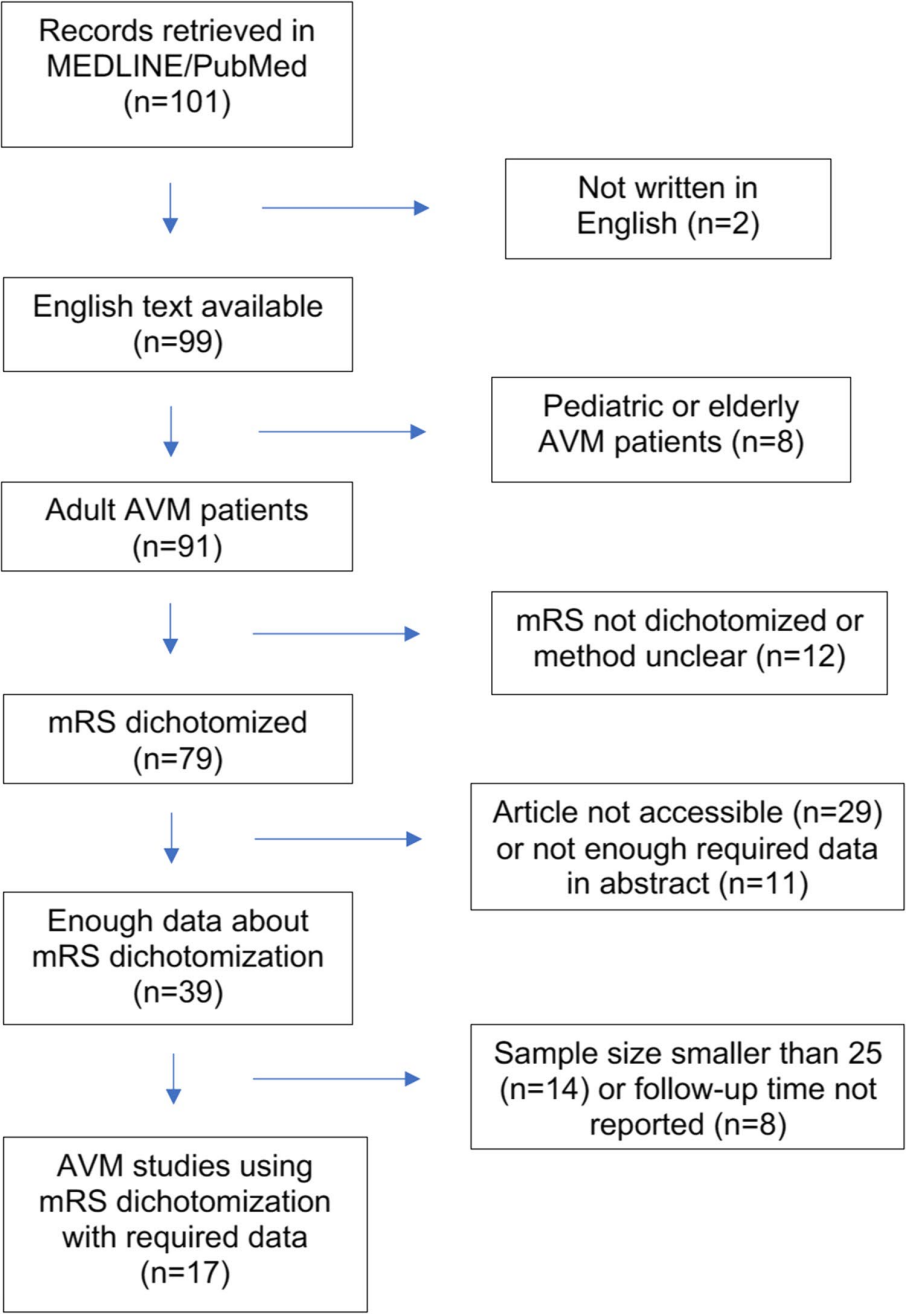
Search protocol for the literature review. The figure illustrates the exclusion and inclusion criteria for the review

We explored the previous 5 years (2015 January to 2020 August) of brain AVM follow-up studies on adult patients using search terms “AVM,” “arteriovenous malformation,” “mRS,” “modified Rankin Scale,” and “functional outcome” on MEDLINE/PubMed. The search was conducted on 2 August 2020. We included studies which were published in English, had used mRS dichotomization in outcome evaluation, and reported the mRS cut point, mean follow-up time, and sample size. We excluded studies on pediatric and elderly patients with AVMs, studies which did not report follow-up time, number of patients, studies which did not use dichotomization of mRS and if the sample size was smaller than 20 patients. These criteria were evaluated first based on abstracts; however, if uncertainty existed based on this, the article was pulled for full-text review. The detailed protocol of the search is illustrated in Fig. 1.

## Results

The demographics and follow-up time data for all the patients (*n* = 323) in each mRS grade are given in Table 1. All participants had at least 1 year from their admission to answering the survey (follow-up time). The mean follow-up time from admission to the questionnaire was 19.4 years (SD =  ± 13.8 years).

**Table 1:** Demographics. Demographic characteristics of the study cohort of 323 adult patients with brain arteriovenous malformation

	Females	AVM fully occluded	Mean age in 2016 (years)	Mean age during admission (years)	Mean follow-up time (years)	Follow-up time range (years)
mRS 0						
* N* = 154	61 (40%)	128 (82%)	52.5 SD = ± 16.1	32.4 SD = ± 15.3	18.9 SD = ± 13.0	1.7–63
mRS 1						
* N* = 78	44 (56%)	62 (80%)	50.9 SD = ± 16.4	35.8 SD = ± 17.4	22.0 SD = ± 15.6	1.2–62
mRS 2						
* N* = 39	20 (51%)	28 (72%)	56.5 SD = ± 16.5	32.1 SD = ± 13.8	16.5 SD = ± 11.8	1.3–52
mRS 3						
* N* = 32	21 (66%)	28 (88%)	56.8 SD = ± 15.0	38.0 SD = ± 18.8	19.6 SD = ± 11.7	1.3–50
mRS 4						
* N* = 20	13 (62%)	18 (86%)	67.1 SD = ± 8.9	48.7 SD = ± 18.3	23.5 SD = ± 20.0	1.4–59
Total						
* N* = 323	159 (49%)	262 (81%)	54.0 SD = ± 16.2	34.7 SD = ± 16.6	19.4 SD = ± 13.8	1.2–63

### 15D score comparisons: mRS grades and the general population

**Fig. 2:**
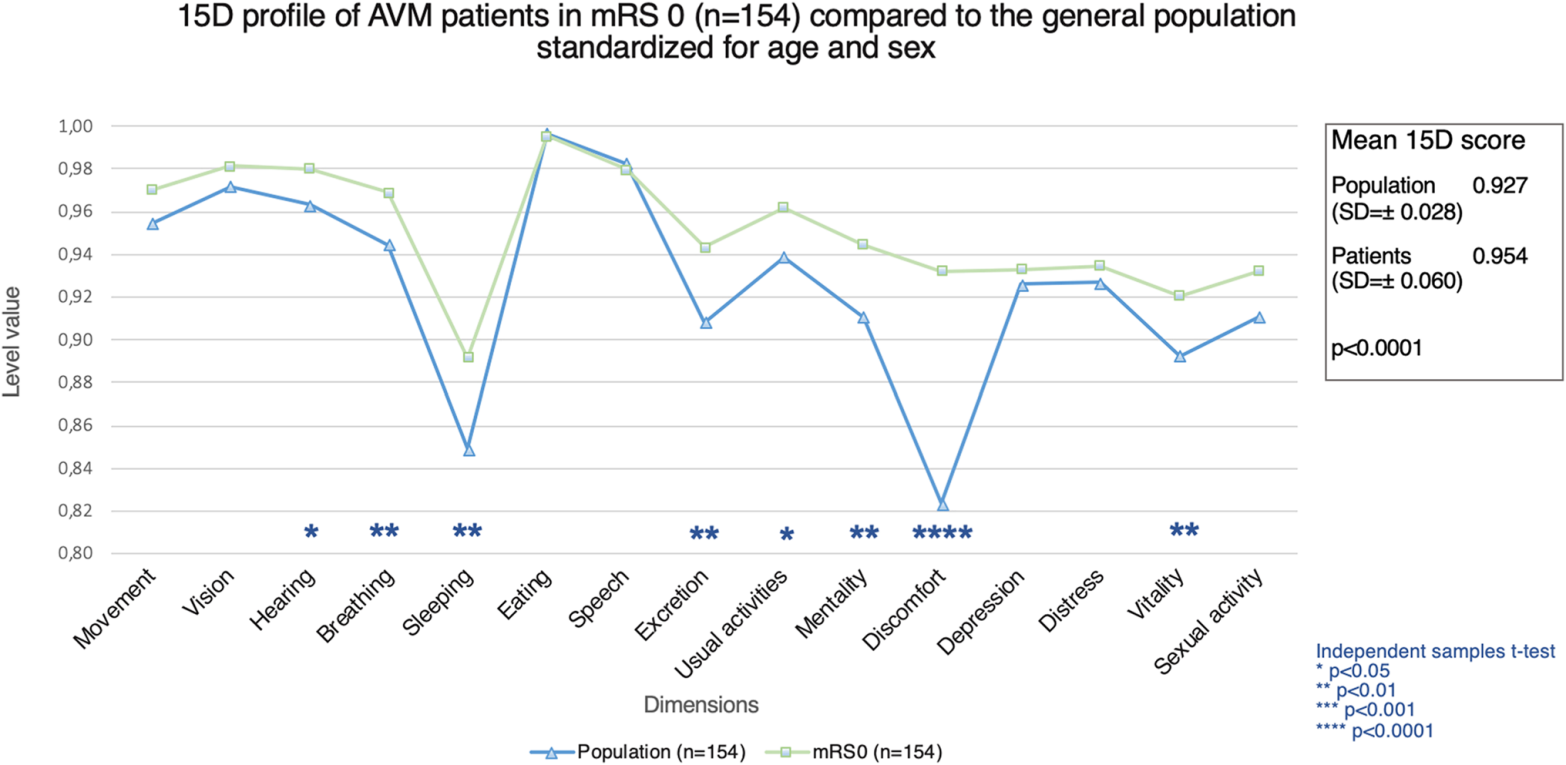
HRQoL comparison of AVM patients in mRS 0 to the general population. The figure illustrates the HRQoL profiles for AVM patients in mRS 0 (green line) at last follow-up (mean = 18.9 years, SD =  ± 13 years) compared to age- and sex-standardized general population (blue line)

**Fig. 3:**
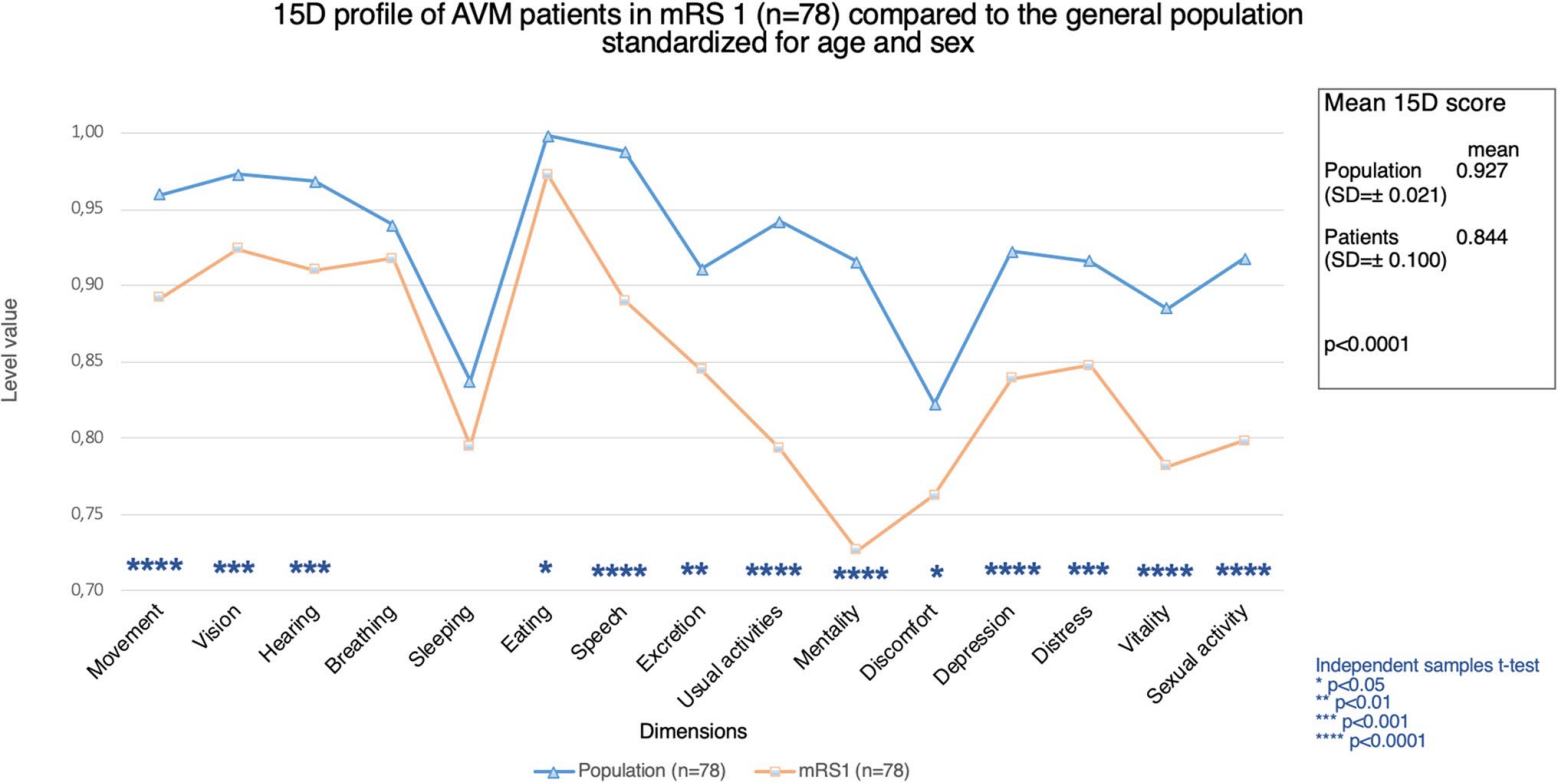
HRQoL comparison of AVM patients in mRS 1 to the general population. The figure illustrates the HRQoL profiles for AVM patients in mRS 1 (green line) at last follow-up (mean = 22.0 years, SD =  ± 15.6 years) compared to age- and sex-standardized general population (blue line)

**Fig. 4:**
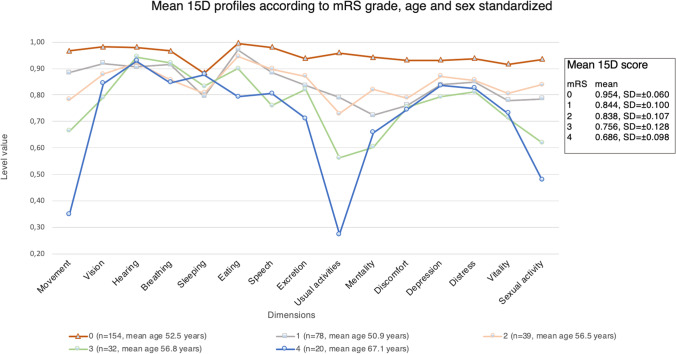
HRQoL comparison between mRS grades, age and sex standardized. The figure includes all the mRS grades and their HRQoL profiles. Profiles are drawn with age- and sex-standardized values. The estimated mean 15D values for this dimension were 0.968 (95% CI = 0.946–0.991) for mRS 0 patients; 0.885 (95% CI = 0.853–0.916) for mRS 1; 0.783 (95% CI = 0.738–0.827) for mRS 2; 0.662 (95% CI = 0.613–0.711) for mRS 3, and 0.311 (95% CI = 0.246–0.376) for mRS 4. In the dimension of usual activities, all the grades, except mRS 1 and 2, differed statistically significantly from one another: the estimated means for this dimension were 0.959 (95% CI = 0.932–0.986) for mRS 0 patients; 0.790 (95% CI = 0.753–0.828) for mRS 1; 0.730 (95% CI = 0.676–0.783) for mRS 2; 0.561 (95% CI = 0.503–0.620) for mRS 3 and 0.283 (95% CI = 0.206–0.360) for mRS 4

Patients in mRS 0 (*n* = 154, mean 15D score = 0.954, SD =  ± 0.060) had better total HRQoL compared to age- and sex-standardized general population (mean = 0.927, SD =  ± 0.028), *p* < 0.0001 (Fig. 2). Figures 3 and 4 present the profiles for mRS grades 0 and 1, respectively, compared to the general population using age and sex standardization. The mean total 15D score for patients in mRS 1 (*n* = 78, mean = 0.844, SD =  ± 0.100) was worse than that of the age- and sex-standardized population controls (mean = 0.927, SD =  ± 0.021), *p* < 0.0001 (Fig. 2).

### 15D score comparison: mRS grades compared to each other using age and sex standardization

**Fig. 5:**
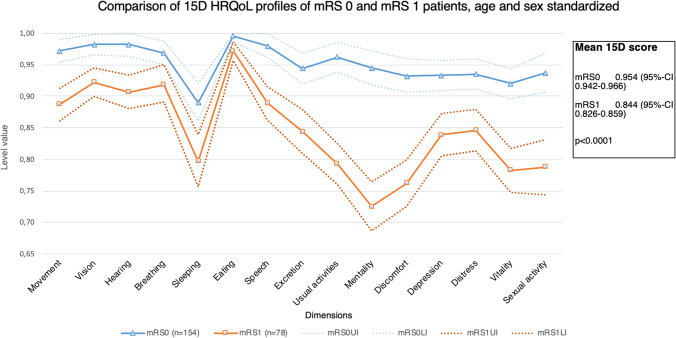
HRQoL comparison between patients in mRS 0 and mRS 1, age and sex standardized. The figure illustrates the 15D profiles for mRS 0 and mRS 1 patients with 95% CIs. The only dimensions with an insignificant difference were eating and breathing. The index score for mRS 0 was 0.954 (95% CI 0.942–0.966) and for mRS1 0.844 (95% CI 0.826–0.859)

All the mRS grades differed significantly in their mean total 15D scores, except mRS 1 (mean = 0.844, 95% CI = 0.826–0.859) and mRS 2 (mean = 0.838, 95% CI = 0.813–0.868). When comparing all the grades to one another, the only HRQoL dimension with distinct values for each mRS grade was mobility (Fig. 3). Patients in mRS 0 compared to mRS 1 patients differed in all other HRQoL dimensions except breathing and eating (Figs. 4 and 5). The total scores were significantly different in pairwise comparison, with index score of 0.954 (95% CI = 0.942–0.966) for mRS 0 patients and 0.844 (95% CI = 0.826–0.859) for mRS 1.

### Literature review

All the 17 AVM follow-up studies using mRS dichotomization published within the previous 5 years are represented in Table 2. Nine studies (52.9%) categorized favorable outcomes as mRS 0–2 and unfavorable as mRS 3–5 [12, 15, 18, 20, 21, 23, 28, 37, 40]. The rest (47.1%) used the lower cut point of mRS 1 [6, 14, 16, 17, 24, 30, 31, 35]. All studies with high grade or brainstem AVMs used the higher cut point [12, 18, 21]. In the studies using the cut point of mRS 2, the mean follow-up time was 3.4 years (SD =  ± 3.1 years) and for the cut point mRS 1 studies 2.4 years (SD =  ± 1.9 years).

**Table 2:** AVM outcome studies using mRS dichotomization. Neurosurgical studies of patients with brain arteriovenous malformation which have been published after 2015 and use mRS dichotomization in their outcome assessment

Author (year)	Mean follow-up time (years)	Favorable mRS	Sample size	AVM lesion characteristics
Wang et al. (2020) [18]	4.5	0–2	258	Low-grade, SM† I–II AVMs
Pulli et al. (2019) [20]	5.0	0–1	318	Cerebral AVMs
Iosif et al. (2019) [17]	0.5	0–2	73	Low-grade AVMs
Kocer et al. (2019) [16]	0.5	0–2	31	High-grade, SM† III–V AVMs
Jean et al. (2019) [21]	1.6	0–1	86	90% lobar AVMs
Madhugiri et al. (2018) [15]	4.0	0–2	39	Brainstem AVMs
Hung et al. (2018) [22]	3.0	0–1	137	SM II AVMs
Pohjola et al. (2018) [14]	9.7	0–2	38	Posterior fossa AVMs
Mascitelli et al. (2018) [13]	2.0	0–2	241	Eloquently located AVMs
Lin et al. (2017) [12]	1.6	0–2	184	39% eloquently located AVMs
Schramm et al. (2017) [23]	5.3	0–1	288	Cerebral AVMs
Morgan et al. 2017 [24]	1.0	0–1	675	SM I–III AVMs
Bervini et al. (2017) [25]	1.0	0–1	769	87% supratentorial
Tong et al. (2017) [19]	6.4	0–2	181	Cerebellar AVMs
Javadpour et al. (2016) [26]	0.5	0–1	45	Unruptured AVMs
Potts et al. (2015) [27]	1.7	0–1	232	SM I–II AVMs
Han et al. (2015) [11]	1.3	0–2	27	Brainstem AVMs

^†^Spetzler-Martin grade

## Discussion

### Key results

We observed that in the long-term follow-up, patients in mRS 0 had a considerably better total HRQoL when compared to patients in mRS 1. To strengthen this point, patients in mRS 0, unlike those in all the other grades, had even better subjective total HRQoL than the age- and sex-standardized general population. Patients in mRS 1 and mRS 2 had a very similar HRQoL profile, even though they are often characterized as being very different by setting the mRS cut point between them, as delineated by the literature review. The higher cut point between 2 and 3 was equally common in modern AVM research as the lower one, and all the studies with worse expected results used the higher cut point [12, 18, 21].

### HRQoL in mRS 0 patients

According to our results, patients in mRS 0 formed a distinctive group with superior HRQoL compared to the rest. This finding is against the recent findings of two meta-analyses using the EQ-5D instrument on stroke patients [32, 41]. In these studies, patients in mRS 0 and 1 formed the most closely associated grades according to utility weights. The contradicting finding of our study could be owing to the different HRQoL instruments used. 15D has in comparison to EQ-5D and many other commonly used instruments illustrated a better detection rate for the psychological dimensions [27]. Furthermore, in comparison to EQ-5D, 15D has a lower ceiling effect and a higher detection rate for change [13]. Having a high ceiling effect means that the improvement in HRQoL with patients in the best possible health states might remain undetected because the scale runs out. Thus, EQ-5D could overreact to better than average health states by producing full index scores too easily [13]. Our findings could also illustrate a difference in the pathophysiological nature of the diseases between the mostly ischemic stroke patients in the aforementioned meta-analyses and the AVM patients in our study. Regarding the comparison to the general population, it should be noted that a random population sample contains participants with worse functional capability than mRS 0, which could explain some of the excellent HRQoL values of the mRS 0 AVM patients. Also, it might be difficult to control individuals to estimate their common symptomology compared to patients who have possibly experienced real disabling symptoms. However, even with this, it is fair to say that mRS 0 patients have at least similar subjective HRQoL with the general population or possibly even better.

### Modified Rankin Scale dichotomization

Modified Rankin Scale dichotomization was first applied to an acute stroke trial in the NINDS (National Institute of Neurologic Diseases and Stroke) tissue plasminogen activator trial in 1995, in which the grade was divided into favorable outcome (mRS 0–1) and unfavorable outcome (mRS 2–5) [11], Afterwards, dichotomization has become common also in other neurological and neurosurgical studies, with an ongoing debate about the rightful dichotomy cut point [10]. Between 2007 and 2016, more than half of the published stroke studies had used dichotomy in their statistical analyses [26]. Dichotomization has some statistical advantages [1]. It can lower the error rates of interobserver variability, especially in the mid-range of the scale, and it eases the reporting and interpretation of the results [22]. However, the cut point needs to be carefully chosen. It should support the severity of the illness and the point the effect is anticipated [7]. If the dichotomization cut point is set incorrectly, it can distort the results as it hides the distribution of the individual grades inside the dichotomous class. This loss of information, however, always exists with dichotomization, unrelatedly of the correctness of the cut point [9]. Often, the cut point is chosen to be set at the sample median. Despite often regarded to be an increasing factor of statistical power, a so-called median split often actually reduces statistical power and can lead to falsely significant results [3]. To avoid these issues, numerous non-dichotomic statistical methods have been developed which rather than compare two fixed classes to one another, attempt to better take into account the movement across the whole scale [5, 25]. These ordinal analyses of mRS have proved more reliable than dichotomy in reporting the outcomes and cost-analyses of stroke patients [10, 34]. Even though these alternative methods are available, dichotomization exists in our research and as many hallmark studies have used it, a deeper understanding of the dichotomy and the individual grades can improve our interpretation of the existing literature. In the review of AVM studies from the past 5 years, the mRS cut points were equally either between mRS 1 and 2 or between 2 and 3. Interestingly, the studies which used the higher cut point were those with difficult AVMs and supposedly worse outcomes. It is tempting to speculate on the possible reasons behind the decision of a higher cut point. With a worse expected outcome of patients with for example brainstem AVM compared to cortical AVMs, it seems reasonable to choose a higher cut point. Is this reason because with the worse expected results we can loosen the definition of a favorable outcome or because of the inability to catch statistically significant results without a higher cut point?

### What is a favorable mRS?

Dividing outcomes into “good” and “bad” is problematic. A good outcome for some patients might represent unfavorable outcomes for others. For example, after a devastating AVM bleeding leading to severe disability, an improvement in functioning somewhat independently might be perceived as a favorable outcome. In contrast, for a preoperatively asymptomatic AVM patient, a postoperative development of minor symptoms might appear as an unfavorable outcome. As this phenomenon is based on the patient’s subjectivity to the symptoms, it should not substantially affect the evaluation of the traditional mRS, which is an objective functional outcome instrument. Objectivity is needed in research, as it allows transparent comparison of treatment strategies, patients, and institutions. However, when thinking about the quality of care, we cannot be content with only the objective outcome. To understand how the objective mRS translates into HRQoL, we compared the mRS with the 15D results. The differences between the mRS grades were smallest in the psychological dimensions and the biggest differences were illustrated in the dimensions requiring physical capability. This inability to discriminate between the psychological dimensions could be owing to the lack of statistical power; however, in our previous HRQoL report, we were able to distinguish significant differences between the certain subgroups of AVM patients regarding their mental well-being using the 15D and the same patient population, although with smaller sample size [29]. Given that the traditional mRS is a scale of functional outcome and the ability to continue previous usual activities, it was able to illustrate these also in the subjective HRQoL results, apart from the overlapping in mRS 1 and 2 in the ability to continue previous usual activities. Despite being able to illustrate both objective and subjective functional outcomes, the division of good and bad mRS remains difficult. As the goal of treatment is often to improve the patient’s condition or to prevent or stop it from declining, it would seem logical to also set our methodology so that this could be captured, instead of trying to artificially divide the scale into favorable and unfavorable.

### Dichotomization cut points

In the history of mRS dichotomization, two common alternatives exist [10]. First, patients in mRS 1 can still carry on with their previous activities, whereas in mRS 2, they cannot, reasoning the cut in between them [10]. On the other hand, mRS 2 patients can still look after their own affairs without assistance when compared to mRS 3, and therefore, mRS grades ≤ 2 are defined to indicate functional independence, giving an alternative cut point [4, 10]. According to our literature review, both were equally used in neurosurgical studies. One of the dimensions of the HRQoL questionnaire used in our study measures the ability to continue previous usual activities. According to our results, there was no considerable difference in this dimension between the AVM patients in mRS 1 and mRS 2. Also, when comparing mRS 1 AVM patients to the population, there was a serious drop in the ability to continue previous activities, even though by definition they should be able to carry on with their normal lifestyle. These findings support the cut point for functional dependence between the grade mRS 2 and 3. When considering the massive drop in the ability to continue previous activities between mRS 0 and mRS 1 patients, however, it would seem more reasonable to consider mRS 0 patients separately from the rest. Including them in the same dichotomous group with mRS 1 patients could lead to a considerable bias by improving results for mRS 1 patients and worsening them for mRS 0 patients. This effect always exists with dichotomization; however, the magnitude of it in our study sample was rather extreme, as illustrated by the comparison of the patients to the general population. Regarding the ordinal mRS analyses, it should be noted that the gap of HRQoL between mRS 0 and mRS 1 could be substantially greater than the gap between the remaining grades. As already discussed, these findings differ from the UW-mRS stroke studies which regard these grades close in utility. A subject of interest for future research is to differentiate whether these findings are owing to the different HRQoL instruments used or the differences in the patient populations.

## Limitations

The variance of the follow-up time created a non-standardized time for outcome assessment, which does not represent the situation in most of the stroke trials, in which the outcome is measured at a certain time point. However, our study better represents many AVM studies, in which the mRS grades are evaluated retrospectively from the clinical records owing to the historical nature of the patient series. The relatively long follow-up time of our study caused a survival bias, which could have affected especially the grades with the poorest outcomes, as the patients with poor initial outcomes might have not survived the years of follow-up. Our results are not generalizable to other than the Finnish population and this is why we encourage other research groups to study their patient cohorts to discover whether this phenomenon is apparent in other populations. The sample size in our study was relatively small, mostly owing to the rarity of AVMs. This increased the uncertainty of our results and the need for similar studies in bigger patient populations.

## Conclusion

Based on our results, mRS illustrates the physical HRQoL dimensions and the ability to continue previous activities also on the subjective level; however, it does not differentiate between the psychological or mental dimensions. In the recent AVM research, it was reasonably common to set the dichotomization cut point between mRS 1 and 2, although we demonstrated that patients in these grades had similar HRQoL. By using an outcome instrument with a lower ceiling effect, we were able to illustrate that AVM patients in mRS 0 have a considerably better subjective HRQoL than the general population controls and mRS 1 patients. This is against the findings in two recent meta-analyses classifying mRS 0 and 1 stroke patients close in utility. This could be explained by the different HRQoL instrument used, the difference in the patient populations, or the limitations of our study discussed earlier. We encourage researchers to study their populations using various HRQoL instruments, as their features vary and can therefore capture differences other instruments cannot. This would widen our understanding of the existing mRS studies and improve future research.
